# Supplementary material for: Shotgun Metagenomic Sequencing Revealed the Prebiotic Potential of a Fruit Juice Drink with Fermentable Fibres in Healthy Humans
Source: Foods. 2023 Jun 25;12(13):2480. doi: 10.3390/foods12132480 (PMC10340277; doi:10.3390/foods12132480)
Supplement: Supplementary file 1 [file foods-12-02480-s001.zip › Supplementary information for MOJUV2.pdf]

Supplemental Table S1. Subject characteristics at baseline (n = 20). Data are Mean ± SD, independent t-test.

Parameter	Group		P value
	AB n = 10	BA n = 10	
Height (cm)	166.25 ± 11.49	171 ± 11.08	.452
Weight (kg)	74.25 ± 10.8	70.50 ± 16.94	.622
BMI	24.75 ± 2.76	23.83 ± 2.13	.498
Age (years)	46.13 ± 9.97	38.17 ± 14.87	.253
Sex (Male/Female)	6F/2M	4F/2M	.761

Supplemental Table S2. Nutritional information of the MOJU Prebiotic Gut Shot per 60ml and 100ml

**Nutrients**	**Per 60ml portion**	**Per 100ml**
**Energy**	134kJ/31.8	224kJ/53kcal
**Fat**<br>***of which saturates***	0.18g<br>0.06g	0.3g<br>0.1g
**Carbohydrate**<br>***of which sugars***	6.6g<br>4.92g	11g<br>8.2g
**Fibre**	3.66g	6.1g
**Protein**	0.3g	0.5g
**Salt**	0g	0g
**Vitamin C**	10.2mg	17mg

Supplemental Table S3: Habitual dietary inulin and oligofructose during the prebiotic supplement Phase. Data are gram per day (Mean ± SD), paired t-test.

Group/Treatment order	Phase	Inulin	Between group p value	Oligofructose	Between group p value
AB	1	4.65 ± 2.68	.156	4.11 ± 2.34	.298
BA	2	6.66 ± 1.92		5.43 ±1.95	

Supplemental Table S4. Habitual dietary inulin and oligofructose during the trial, pooled data. Data are gram per day (Mean ± SD), paired t-test.

Fibre	Prebiotic phase	Placebo phase	Difference	P value
Inulin	6.07 ± 3.01	5.49 ± 2.5	0.578 ± 1.65	.213
Oligofructose	5.12 ±2.58	4.73 ± 2.34	0.384 ± 1.54	.370

Supplemental Table S5. Habitual dietary inulin and oligofructose within-group differences. Data are gram per day (Mean ± SD), paired t-test.

Group	Inulin within-group p value	Difference between phases	Oligofructose within-group p value	Difference between phases
AB	.279	0.84±2.0	.214	0.81±1.69
BA	.612	0.22±1.0	.721	0.19±1.2

Supplemental Table S6. BSS consistency changes (Mean ± SD), paired t-test.

Group	Phase	Pre	Post	Diff	P value
AB	Prebiotic (1)	3.37±0.74	3.87±0.35	0.5±0.75	0.104
AB	Placebo (2)	3.0±1.4	3.62±1.4	0.62±1.5	0.279
BA	Placebo (1)	3.33±0.81	3.50±0.54	0.16±1.16	0.741
BA	Prebiotic (2)	4.0±0.89	4.16±0.75	0.16±1.16	0.741

Supplemental Figure S1. CONSORT (Consolidated Standards of Reporting Trials) flow diagram

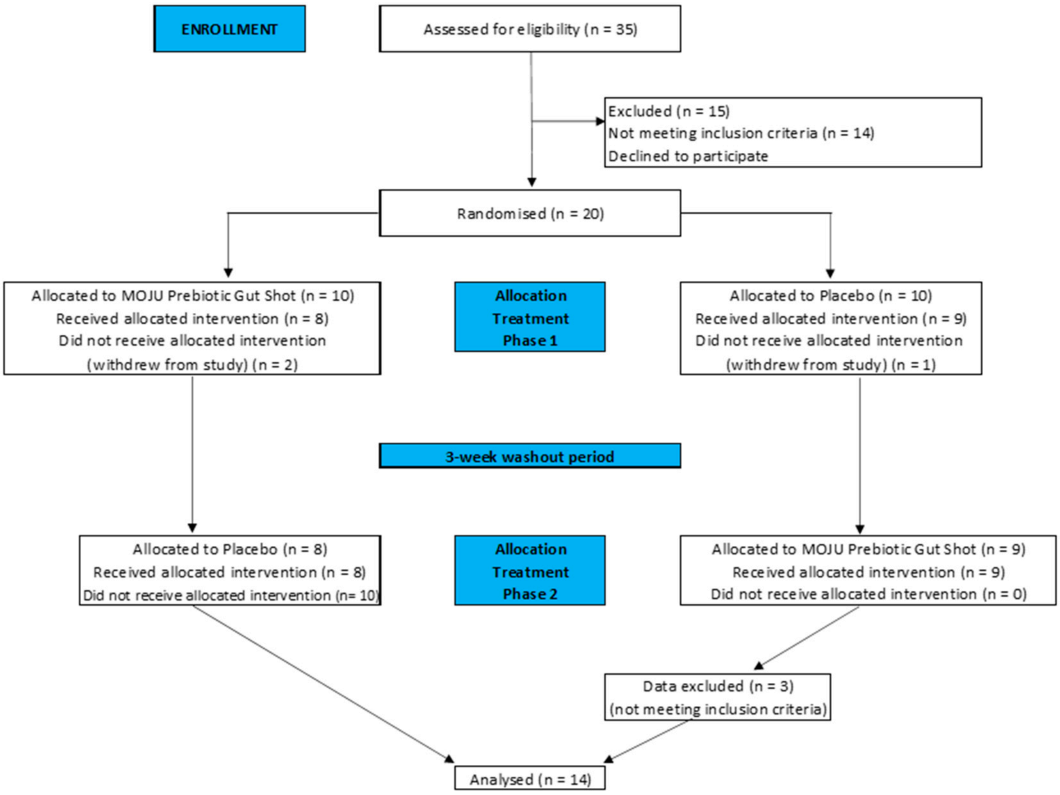

Supplemental Figure S2. Boxplot representation of percentage of reads which pass the quality filtering step, separated, and coloured by subject.

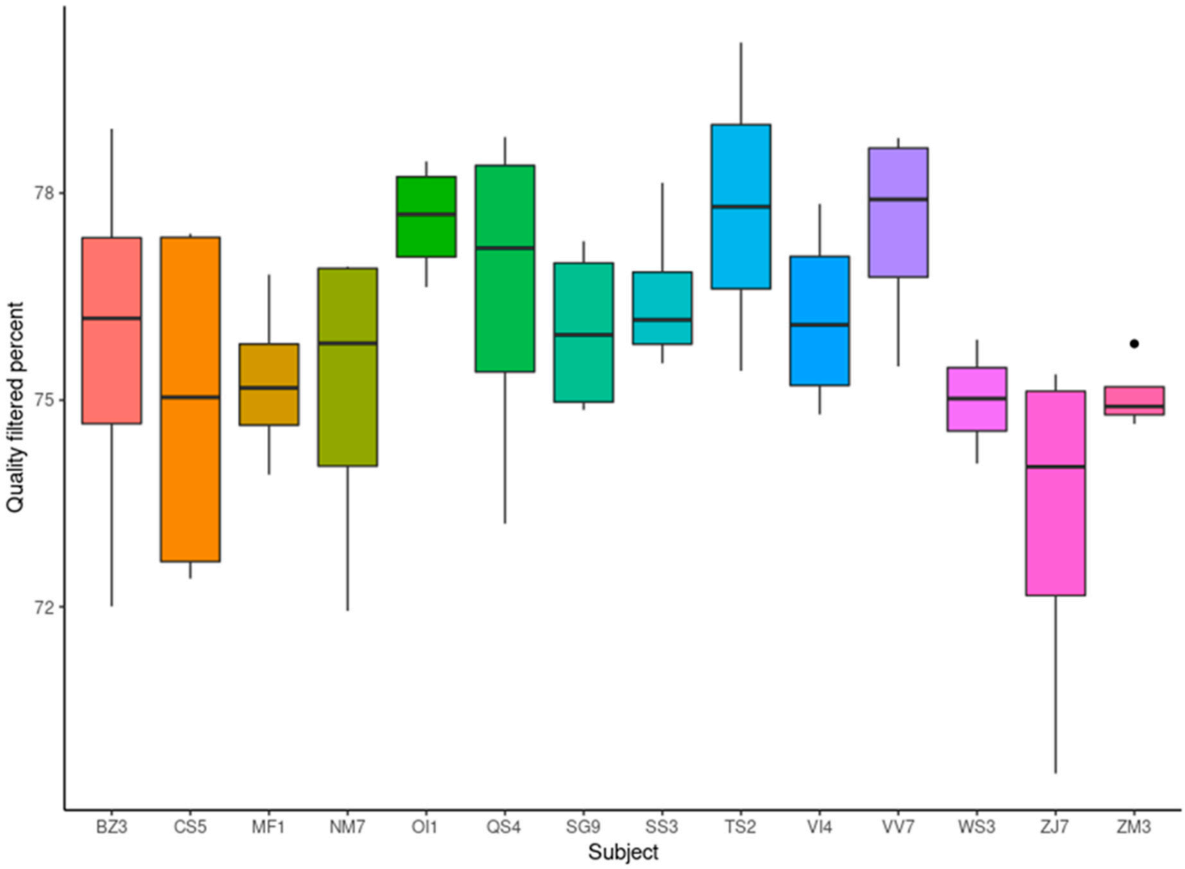

Supplemental Figure S3. Kraken2 percent classification of reads, separated and coloured by participant.

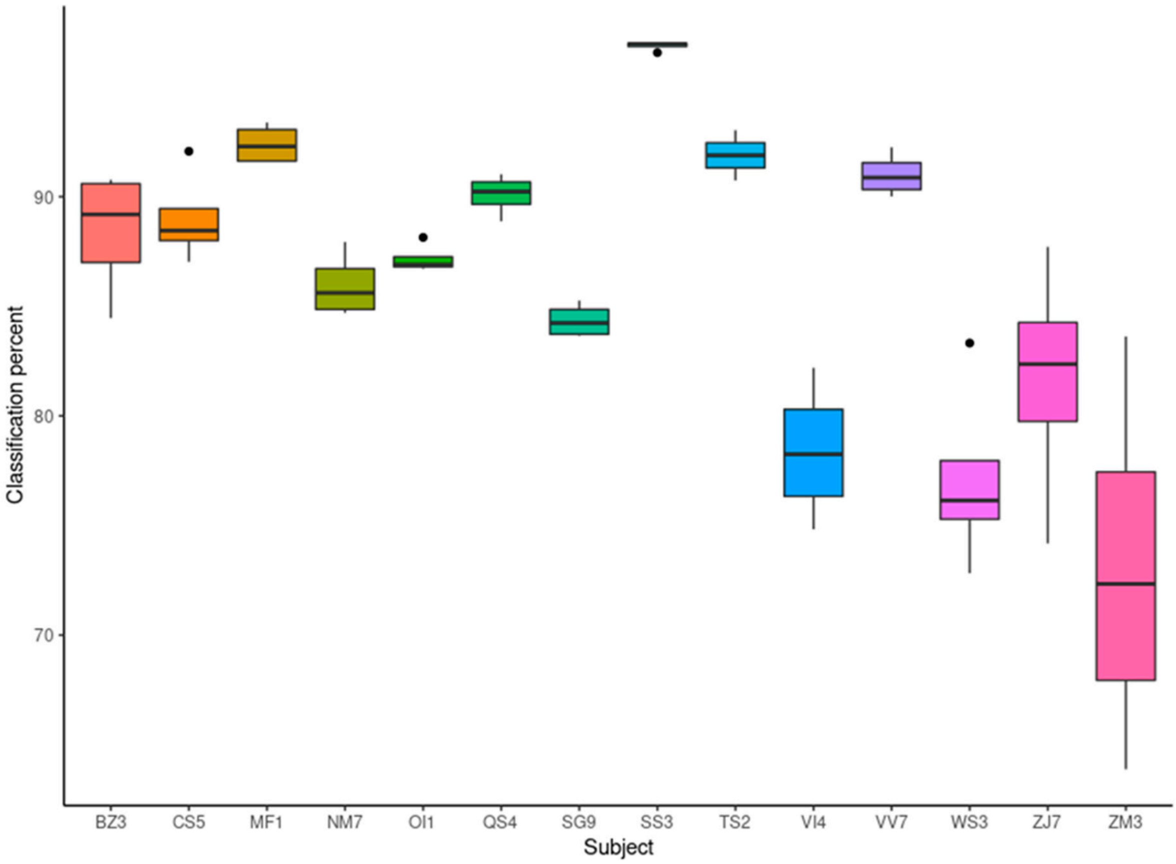

Supplemental Figure S4. Effect of 3-week supplementation of MOJU Prebiotic Shot or placebo (without the fibres) on Depression, as measured by DASS-42. Graphs show within-group changes for each treatment. AB is treatment order prebiotic start (n = 3), and BA is treatment order placebo start (n = 4). Treatment one = MOJU Prebiotic Shot, and treatment two = placebo. Week 1 = baseline, week 2 = end of intervention week 1; week 3 = end of intervention week 2; week 4 = end of intervention week 3

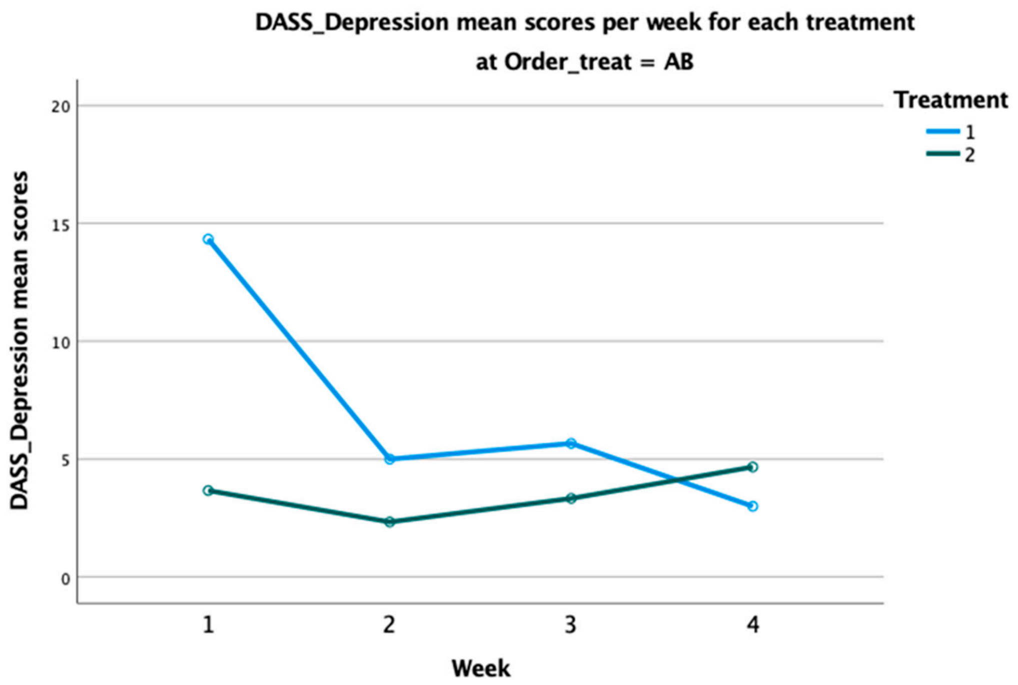

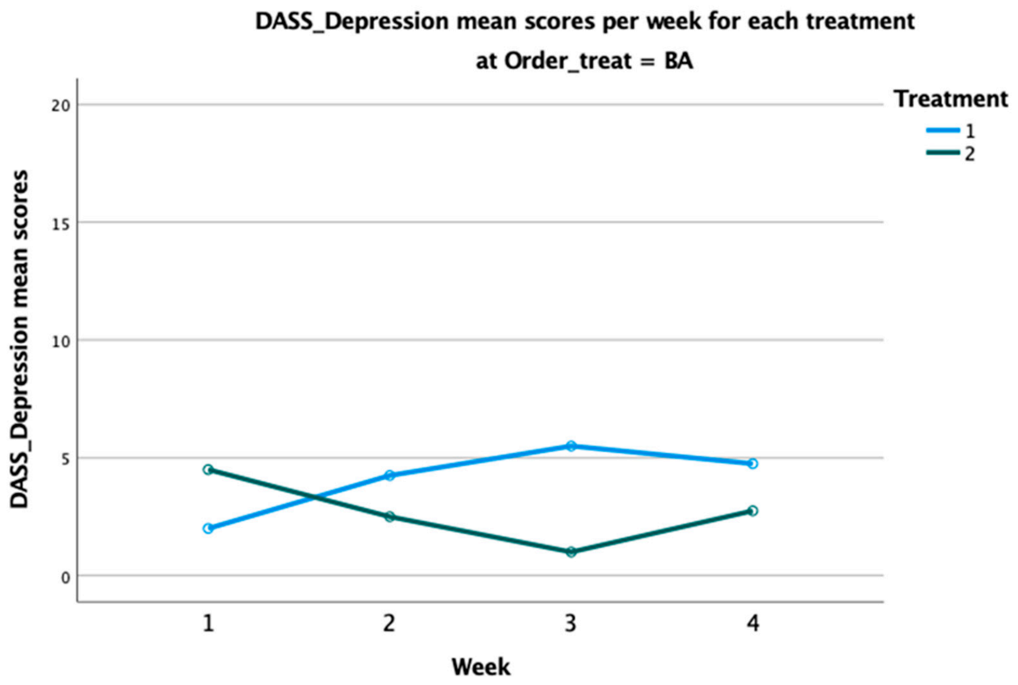

Supplemental Figure S5. Effect of 3-week supplementation of MOJU Prebiotic Shot or placebo (without the fibres) on Anxiety, as measured by DASS-42. Graphs show within-group changes for each treatment. Treatment one = MOJU Prebiotic Shot, and treatment two = placebo. AB is treatment order prebiotic start (n = 3), and BA is treatment order placebo start (n = 4). Week 1 = baseline, week 2 = end of intervention week 1; week 3 = end of intervention week 2; week 4 = end of intervention week 3

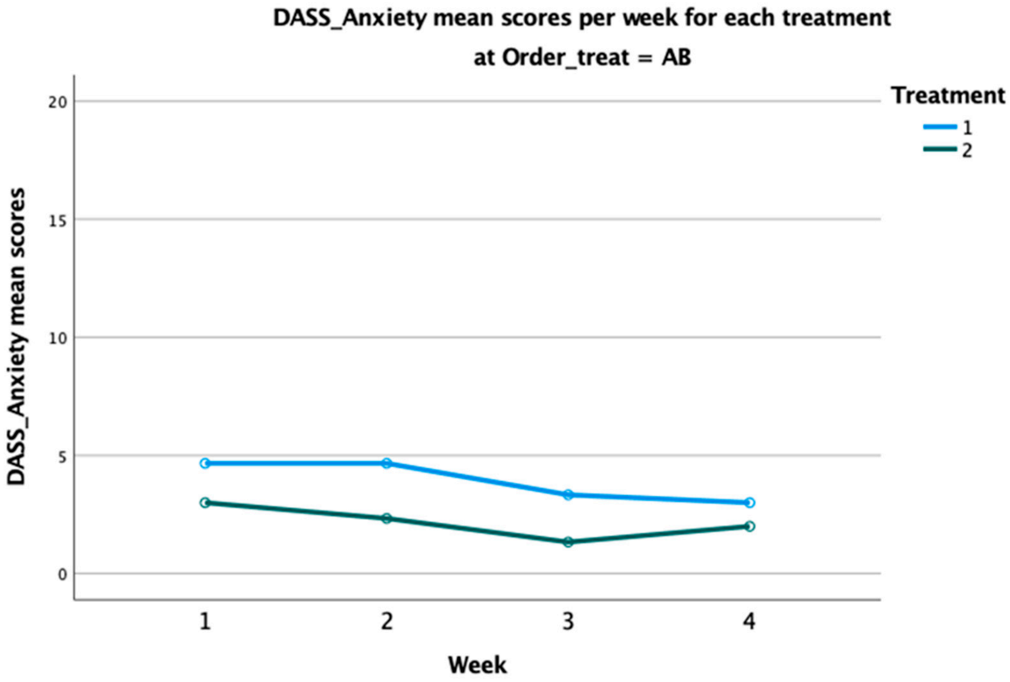

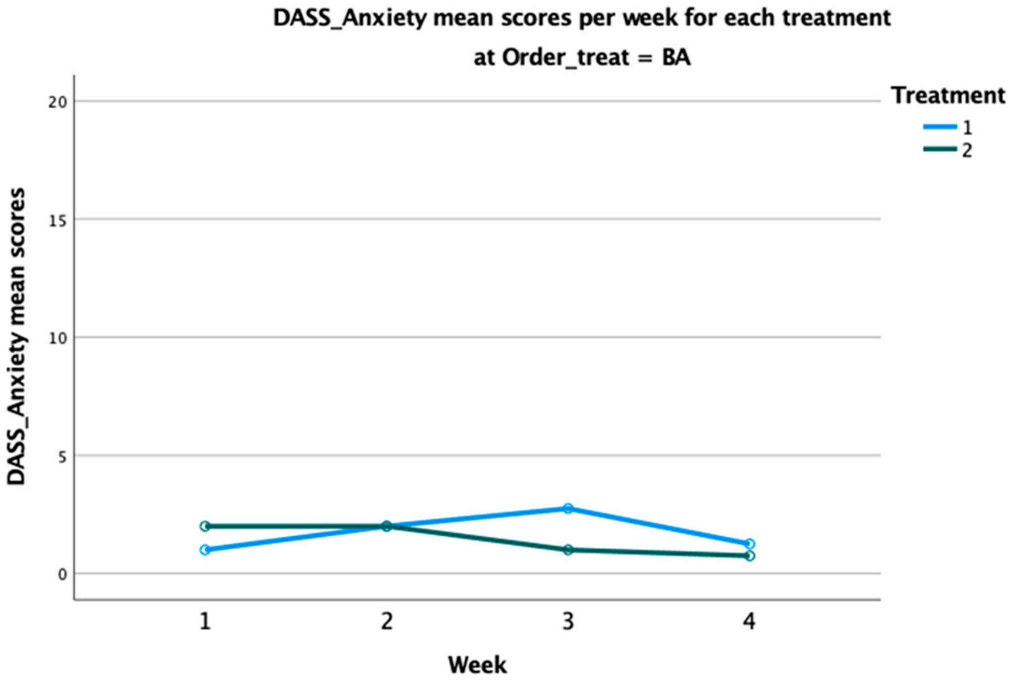

Supplemental Figure S6. Effect of 3-week supplementation of MOJU Prebiotic Shot or placebo (without the fibres) on Stress, as measured by DASS-42. Graphs show within-group changes for each treatment. AB is treatment order prebiotic start (n = 3), and BA is treatment order placebo start (n = 4). Treatment one = MOJU Prebiotic Shot, and treatment two = placebo. Week 1 = baseline, week 2 = end of intervention week 1; week 3 = end of intervention week 2; week 4 = end of intervention week 3

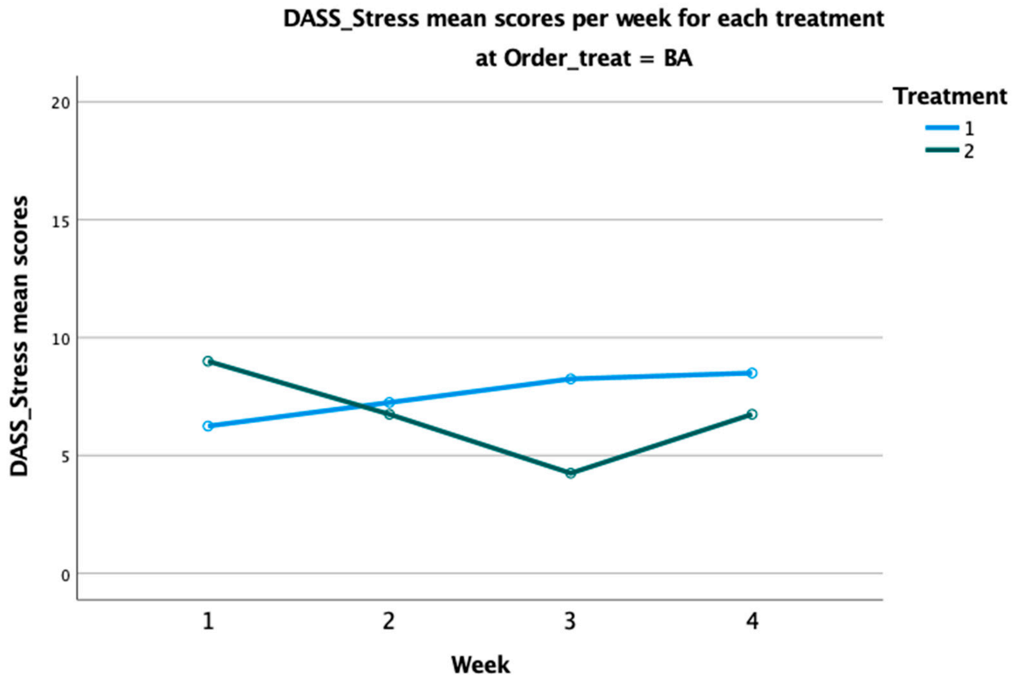

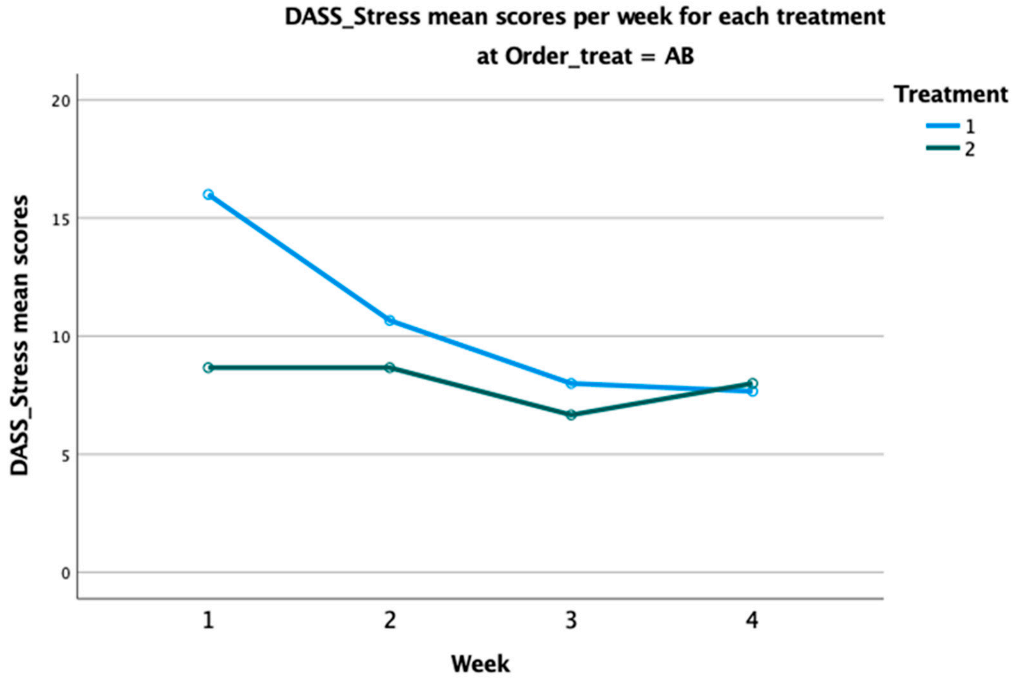

Supplemental Figure S7. Effect of 3-week supplementation of MOJU Probiotic Shot or placebo (without the fibres) on Depression, Anxiety, and Stress as measured by DASS-42. Graphs show between treatment changes for total scores (n = 7). Treatment one = MOJU Prebiotic Shot, and treatment two = placebo. Week 1 = baseline, week 2 = end of intervention week 1; week 3 = end of intervention week 2; week 4 = end of intervention week 3

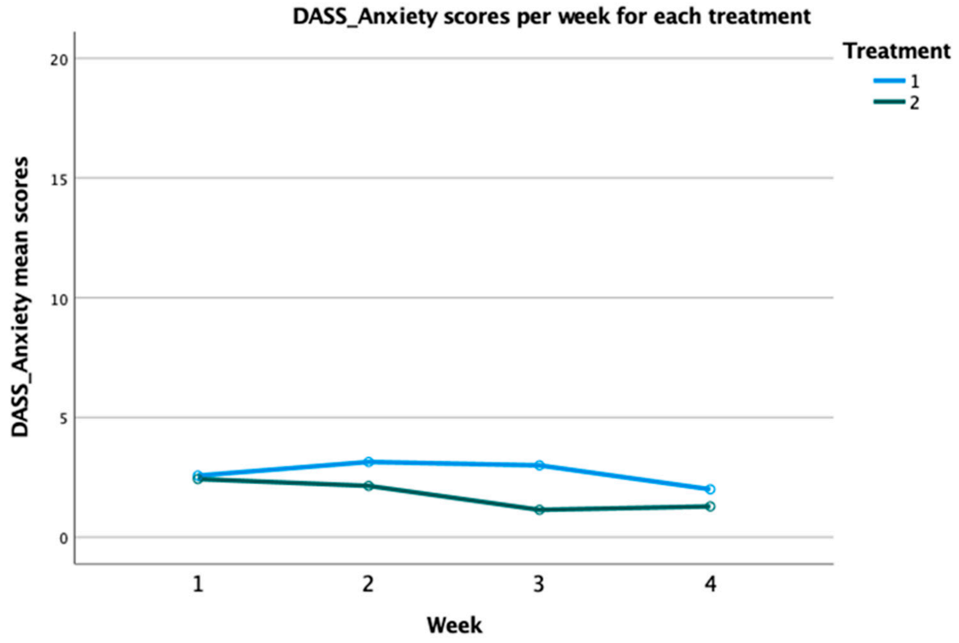

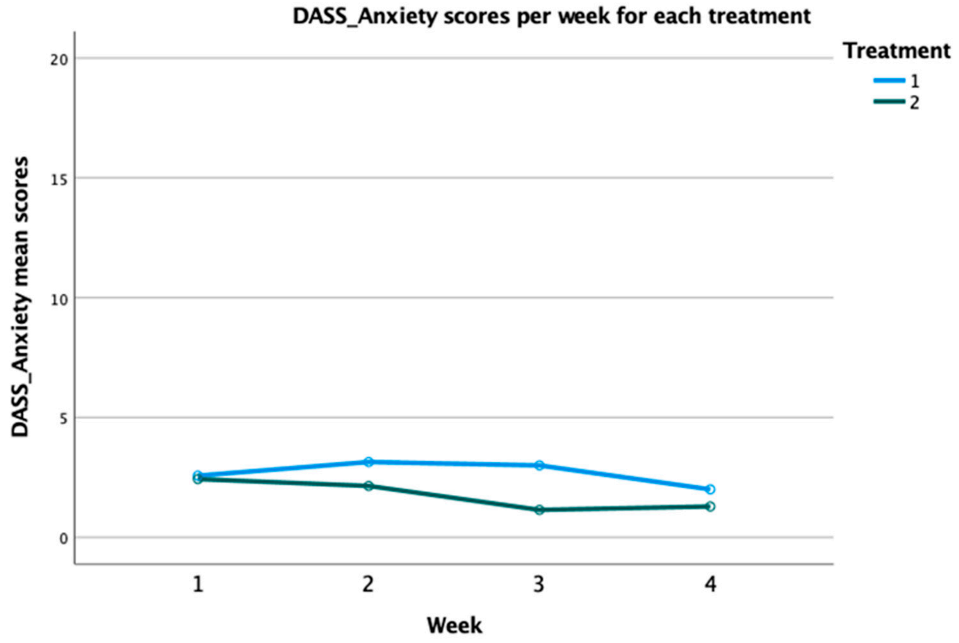

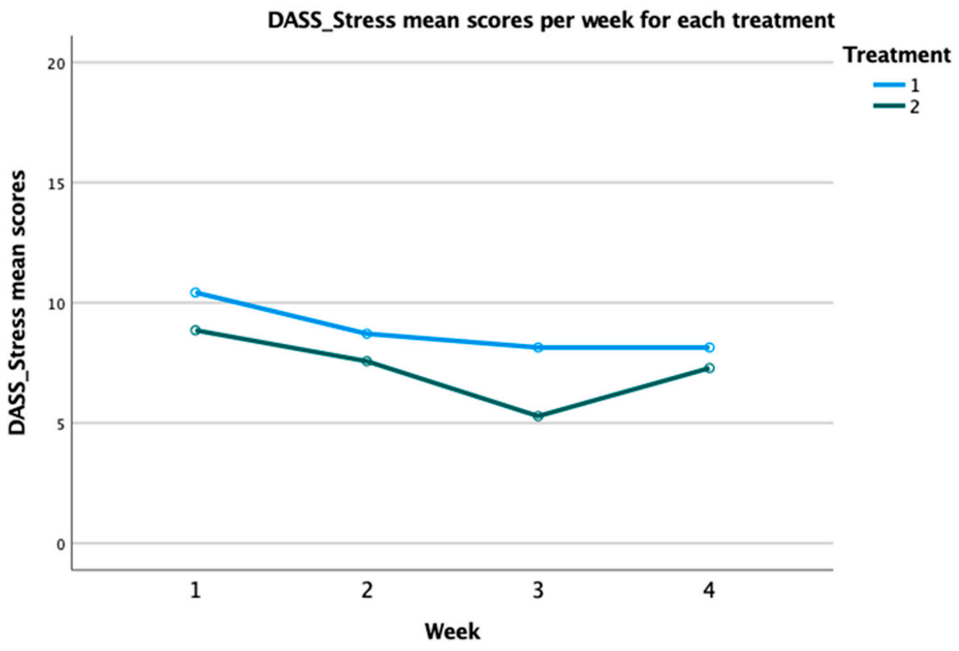

Supplemental Figure S8. Effects of 3-week supplementation of the MOJU Prebiotic Shot or placebo (without the fibres) on gut microbiota at genus level in healthy adults. Stack bar charts represent relative abundance of main phyla for treatment order groups (AB) prebiotic start, and (BA) placebo start.

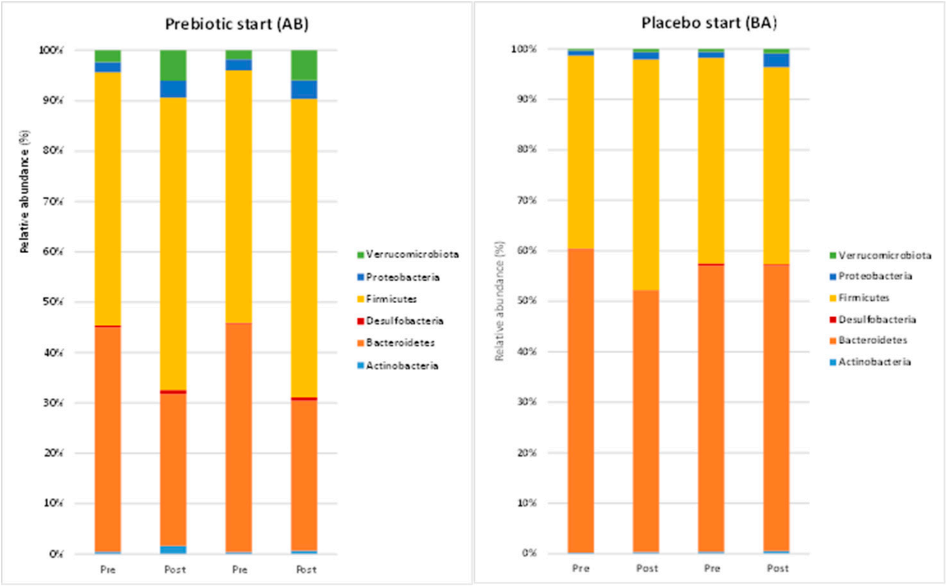

Supplemental Table S7: Results of the multiple linear regression showing no significant effect of age and gender on total relative abundance.

	Unstandardized Coefficients		Standardized Coefficients		
	B	Std. Error	Beta	t-value	p-value
(Constant)	250145.278	624002.594		0.401	0.696
Gender	41264.495	267317.096	0.047	0.154	0.880
age	-6870.349	10051.362	-0.207	-0.684	0.508

Supplemental Table S8: Results of the multiple linear regression showing no significant effect of age and gender on *B. adolescentis* relative abundance.

	Unstandardized Coefficients		Standardized Coefficients		
	B	Std. Error	Beta	t-value	p-value
(Constant)	10097.656	13504.738		0.748	0.470
Gender	-2571.562	5785.308	-0.137	-0.444	0.665
age	-46.244	217.533	-0.066	-0.213	0.836

Supplemental Table S9: Results of the multiple linear regression showing no significant effect of age and gender on CAG81sp900066785 relative abundance.

	Unstandardized Coefficients		Standardized Coefficients		
	B	Std. Error	Beta	t-value	p-value
(Constant)	-1025.335	1995.321		-0.514	0.618
Gender	864.597	854.777	0.299	1.011	0.334
age	-5.069	32.140	-0.047	-0.158	0.878
